# Impact of performance in a mandatory postgraduate surgical examination on selection into specialty training

**DOI:** 10.1002/bjs5.7

**Published:** 2017-08-29

**Authors:** D. S. G. Scrimgeour, J. Cleland, A. J. Lee, G. Griffiths, A. J. McKinley, C. Marx, P. A. Brennan

**Affiliations:** ^1^ Centre For Healthcare Education Research and Innovation University of Aberdeen Aberdeen UK; ^2^ Department of Medical Statistics University of Aberdeen Aberdeen UK; ^3^ Department of Colorectal Surgery Aberdeen Royal Infirmary Aberdeen UK; ^4^ Department of Vascular Surgery Ninewells Hospital Dundee UK; ^5^ Intercollegiate Committee for Basic Surgical Examinations London UK; ^6^ Royal College of Surgeons of England London UK

## Abstract

**Background:**

The Intercollegiate Membership of the Royal College of Surgeons (MRCS) examination is undertaken by large numbers of trainees in the UK and internationally as a mandatory step within surgical training. Unlike some high‐stakes medical examinations, the MRCS is yet to be validated. A quantitative study was undertaken to assess its predictive validity by investigating the relationship between MRCS (Parts A and B) and national selection interview scores for general and vascular surgery in the UK.

**Methods:**

Pearson correlation coefficients were used to examine the linear relationship between each assessment, and linear regression analyses were employed to identify potential independent predictors of the national selection score. All UK medical graduates who attempted the interview in 2011–2015 were included.

**Results:**

Some 84·4 per cent of the candidates (1231 of 1458) were matched with MRCS data. There was a significant positive correlation between the first attempt score at Part B of the MRCS examination and the national selection score (r = 0·38, P < 0·001). In multivariable analysis, 17 per cent of variance in the national selection first attempt score was explained by the Part B MRCS score and number of attempts (change in R ^2^ value of 0·10 and 0·07 respectively; P < 0·001). Candidates who required more than two attempts at Part B were predicted to score 8·1 per cent less than equally matched candidates who passed at their first attempt.

**Conclusion:**

This study supports validity of the MRCS examination, and indicates its predictive value regarding entry into specialist training.

## Introduction

**Figure 1:**
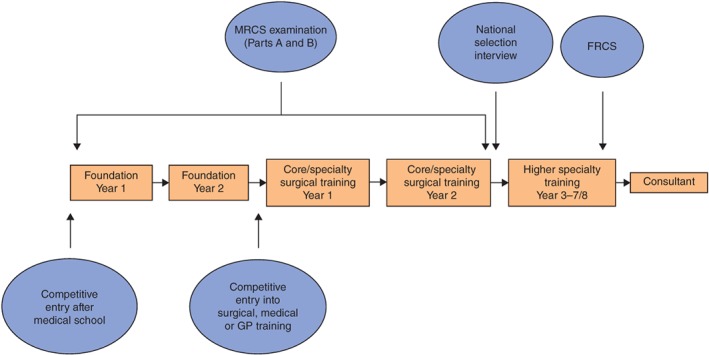
Surgical training pathway in the UK. MRCS, Membership of the Royal College of Surgeons; FRCS, Fellowship of the Royal College of Surgeons; GP, general practice

The Intercollegiate Membership of the Royal College of Surgeons (MRCS) examination is one of the most widely offered postgraduate surgical examinations in the world, with up to 6000 doctors in the UK and overseas taking the examination each year^1^ ^2^. An overview of the medical education and surgical training pathway in the UK is provided in *Fig*. 1. Upon graduating from medical school, newly qualified doctors enter the 2‐year Foundation Programme. Most doctors will continue their training in one of three broad specialist areas: general practice, medicine or surgery. To be considered for higher medical or surgical training, UK doctors must also complete core training and pass the relevant postgraduate examination, MRCS or Membership of the Royal Colleges of Physicians (MRCP). Overviews of the content of the MRCS examination and the specialty training selection process for general and vascular surgery have been described elsewhere^2^ ^3^. The MRCS examination is designed to safeguard patients and ensure high standards for practising surgeons. It is a prerequisite for progression to higher specialty training in the UK, a mandatory examination for all aspiring surgeons who wish to train or work in the UK and a means for overseas candidates to improve their opportunities in their home country^2^ ^4^.

The Intercollegiate Committee for Basic Surgical Examinations (ICBSE), which is responsible for the continued development, quality assurance and standards of MRCS in the UK, produces an annual report highlighting examination reliability^5^. To date, there has been no analysis of the predictive validity of the MRCS examination, and it is not known whether performance at this examination predicts outcomes as a surgeon in training or beyond.

Given that MRCS is a prerequisite for progression to higher specialty training in the UK, it is important to determine the relationship between MRCS examination scores and outcomes in the higher specialty training national selection process. The aim of this study was to examine the predictive validity of MRCS in relation to outcomes in this selection process, and, specifically, whether the results of this mandatory examination can predict how candidates will perform at the national selection interview for a large surgical specialty, general surgery (which included vascular surgery in the UK until 2013).

## Methods

In the absence of a specific ethics committee responsible for postgraduate surgical examinations, both the ICBSE and its Internal Quality Assurance subcommittee, which monitors standards, approved the study.

The sample included all UK medical graduates who had attempted Part B of the MRCS examination from its institution in October 2008 to May 2015. Data were extracted by the lead administrator from the intercollegiate MRCS database held by the Royal College of Surgeons (RCS) of England. Each candidate's Part A score was merged with their Part B score to create a complete MRCS history, which included the self‐declared demographics of sex, ethnicity, first language and date of birth.

Candidates were anonymized by a unique identifier before data were released for analysis. This identifier corresponded to each candidate's General Medical Council number, which could be accessed only by the RCS lead administrator and not by the research team.

All scores (recorded as percentages) for candidates who had attempted the national selection interview for general and vascular surgery since its origin in May 2011 were then cross‐linked with the MRCS database. The national selection first attempt score was used as the main outcome variable.

As the Part B examination underwent changes, three periods were considered: October 2008 to February 2010, May 2010 to October 2012, and February 2013 to present. Each of these periods was considered as one of the variables of interest.

As part of national selection, candidates are divided into three groups at the portfolio station based on how many years they are from graduation: less than 5 years, 5–7 years and more than 7 years. Years from graduation was also considered as a variable of interest.

As older doctors (aged at least 29 years at graduation from medical school) have been shown to have problems with their Annual Review of Competence Progression compared with younger colleagues^6^, this age‐related variable was also considered in the analyses.

Candidates were categorized based on their total number of attempts required to pass each part of the MRCS examination (1, 2, or 3 or more attempts).

### Statistical analysis

All analyses were conducted using SPSS^®^ version 24.0 (IBM, Armonk, New York, USA). Pearson correlation coefficients were used to examine the linear relationship between both parts of the MRCS examination and the national selection first attempt score. The magnitude of correlation was in accordance with Cohen's guidelines^7^ (r = 0·01–0·29, low or weak correlation; r = 0·30–0·49, moderate correlation; r ≥ 0·50, strong correlation). An independent‐samples t test or ANOVA was used to examine the relationship between MRCS and national selection scores and demographic variables, dependent on the number of categories being compared.

Linear regression analysis was used to identify potential independent predictors of the national selection score. All potential predictors with P < 0·100 in univariable analysis were entered simultaneously into the regression models. Any variable with P > 0·050 in the full model was subsequently removed until only statistically significant predictors remained in each model.

Stepwise linear regression analysis was used to measure the change in R^2^ when single predictors were added to the model in order to assess the magnitude of the contribution of new predictors in explaining variance in national selection score.

There were no violations of the assumptions for any of the regression models and no collinearity was evident in the data.

## Results

**Figure 2:**
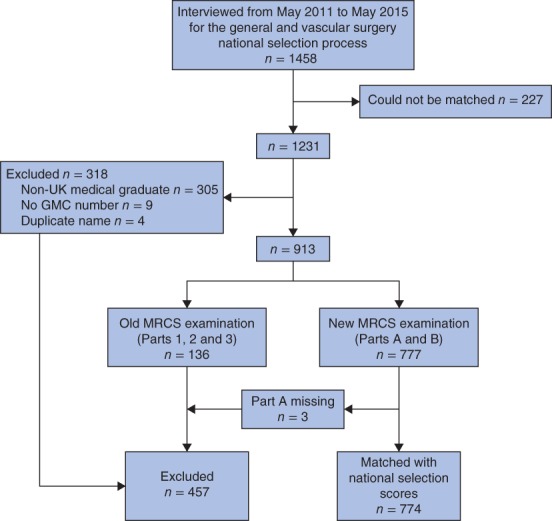
Flow diagram of surgical trainees in the study. GMC, General Medical Council; MRCS, Membership of the Royal College of Surgeons

A total of 1231 (84·4 per cent) of 1458 candidates long‐listed for the general and vascular surgery national selection process in the UK from May 2011 to May 2015 were matched with MRCS data (Fig. 2). Some 454 candidates could not be included: 305 were non‐UK graduates, nine had no GMC number available, four were duplicate entries and 136 had sat the MRCS examination in place before 2008. Of the remaining 777 candidates, three had incomplete Part A data available and were also excluded, so that 774 candidates were matched on MRCS and national selection data, and entered into the analysis.

The majority of national selection candidates were: white British (353 of 598, 59·0 per cent), men (440 of 772, 57·0 per cent), spoke English as their first language (571 of 611, 93·5 per cent) and graduated from medical school before the age of 29 years (734 of 772, 95·1 per cent). Only 13 (1·7 per cent) of 773 candidates attempted the national selection interview for the first time more than 7 years after graduating from medical school. More than half (423 of 773, 54·7 per cent) of the UK graduates in the cohort had passed the Part B MRCS examination between May 2010 and October 2012 (*Table* 1).

**Table 1:** National selection descriptive statistics and the relationship between demographic variables and national selection first attempt score for general and vascular surgery

	National selection descriptive statistics (n = 774)*	National selection first attempt score (%)†	Test statistic‡	d.f.	P
Age (years)†	29·18(2·68)				
Sex			0·25§	754	0·804
M	440 (57·0)	73·27(9·19)			
F	332 (43·0)	73·43(8·01)			
Missing	2				
Ethnicity			6·22	4	< 0·001
White British	353 (59·0)	74·92(7·52)			
Asian	156 (26·1)	71·32(8·14)			
Black	20 (3·3)	72·09(9·67)			
Other	69 (11·5)	71·43(10·33)			
Missing	176				
First language			2·70§	609	0·007
English	571 (93·5)	73·72(8·11)			
Not English	40 (6·5)	70·10(9·37)			
Missing	163				
Mature medical graduate			1·26§	770	0·209
No (age < 29 years)	734 (95·1)	73·42(8·67)			
Yes (≥ 29 years)	38 (4·9)	71·61(8·99)			
Missing	2				
No. of Part A MRCS attempts			4·64	2	0·010
1	598 (77·3)	73·85(8·69)			
2	90 (11·6)	71·14(8·45)			
≥ 3	86 (11·1)	72·24(8·59)			
No. of Part B MRCS attempts			27·04	2	< 0·001
1	581 (75·1)	74·50(7·93)			
2	150 (19·4)	71·01(10·10)			
≥ 3	43 (5·6)	66·10(8·33)			
Years from graduation			1·64	2	0·196
< 5	668 (86·4)	73·49(8·61)			
5–7	92 (11·9)	72·86(9·31)			
> 7	13 (1·7)	69·32(8·07)			
Missing	1				
Part B examination date			1·20	2	0·301
October 2008 to February 2010	111 (14·4)	73·14(11·07)			
May 2010 to October 2012	423 (54·7)	73·02(8·41)			
February 2013 to present	239 (30·9)	74·09(7·91)			
Missing	1				

Values in parentheses are

* percentages and

† mean(s.d.). n.a., Not applicable; MRCS, Membership of the Royal College of Surgeons.

‡ ANOVA, except

§ t test.

Approximately three‐quarters of national selection candidates had passed Part A (598 of 774, 77·3 per cent) and Part B (581 of 774, 75·1 per cent) of the MRCS examination at the first attempt. There were significant, weak correlations between Part A first and passing attempt scores and the national selection first attempt score (*r* = 0·19 and 0·20 respectively; *P* < 0·001). The correlations between Part B first and passing attempt scores and the national selection score were stronger (*r* = 0·38 and 0·30 respectively; *P* < 0·001) (*Table* 2).

**Table 2:** Correlations between Membership of the Royal College of Surgeons examination scores and national selection first attempt score for general and vascular surgery

	National selection first attempt (*n* = 774)
Part A	
First attempt	0·19
Passing attempt	0·20
Part B	
First attempt	0·38
Passing attempt	0·30

Values are Pearson correlation coefficients; all are significant at *P* < 0·001 level.

The relationship between demographic factors and the number of attempts at each part of the MRCS examination, and how they affected the national selection first attempt score, are presented in *Table* 1.

In univariable analysis, there were significant differences in national selection first attempt score between the variables: number of attempts at Part A MRCS (*t* = 4·64, 2 d.f., *P* = 0·010), number of attempts at Part B MRCS (*t* = 27·04, 2 d.f., *P* < 0·001), ethnicity (*t* = 6·22, 4 d.f., *P* < 0·001) and first language (*t* = 2·70, 609 d.f., *P* = 0·007). As these variables were all below the 10 per cent significance level in univariable analysis, they were included in the multivariable analysis to identify independent predictors of national selection score for general and vascular surgery (*Table* 3).

**Table 3:** Multivariable linear regression analysis of predictors of national selection first attempt score for general and vascular surgery

	Unstandardized coefficients		
	B	s.e.	*P*
Constant*	69·67	0·97	< 0·001
How well candidates passed Part B MRCS (% above pass mark)†	0·28	0·04	< 0·001
No. of Part B MRCS attempts			
1	Reference		
2	−2·93	0·79	< 0·001
≥ 3	−8·11	1·31	< 0·001
Ethnicity			
White British	Reference		
Asian	−1·77	0·75	0·019
Black	−1·08	1·74	0·536
Other	−1·30	1·02	0·201

* Maximum predicted first attempt score, calculated using the reference variables (a white candidate who passed Part B Membership of the Royal College of Surgeons (MRCS) at the first attempt is expected to score 69·67 per cent at the national selection interview for general and vascular surgery).

† A candidate who just achieved the pass mark would have a value of zero. Model: *R* ^2^ = 0·18, *n* = 598.

The Part B MRCS passing attempt score and the number of attempts required to pass Part B were significant predictors of the general and vascular surgery national selection first attempt score (change in *R* ^2^ value of 0·10 and 0·07 respectively; *P* < 0·001). The model also predicted that candidates who required three or more attempts at Part B of the examination would obtain a national selection score 8·1 per cent lower than equally matched candidates who passed Part B at their first attempt. Ethnicity had much less influence on national selection score than number of attempts (change in *R* ^2^ = 0·01, *P* = 0·038), although Asian candidates were predicted to perform slightly worse than white candidates (national selection first attempt score for general and vascular surgery: 67·9 *versus* 69·7 per cent respectively; *P* = 0·019).

## Discussion

Several groups have assessed the predictive validity of other high‐stakes medical examinations, including the United States Medical Licensing Examination^®^ (USMLE^®^)^8^, ^9^, ^10^, the Quebec Licensing Examination^11^, the Medical Council of Canada Qualifying Examination^12^, ^13^, ^14^, the MRCP examination^15^, ^16^, ^17^ and Membership of the Royal College of General Practitioners examination^15^. Performance in these examinations predicts performance in clinical practice and other medical assessment processes^11^ ^18^, ^19^. For example, each part of the MRCP examination predicts the next^17^, and candidates who score higher in all parts of the MRCP examination do better in workplace‐based assessments than those who underperform^16^. Similarly, USMLE^®^ scores predict performance in the American Board of Surgery In‐Training Examination among general surgery residents^20^, and higher USMLE^®^ scores have been associated with higher pass rates in the general surgery board examination^10^. This may explain why surgical programme directors in the USA regard USMLE^®^ performance as the most important factor for preliminary screening of general surgery residents^21^.

In keeping with these observations, the present study has identified that performance in Part B of the MRCS examination predicts 17 per cent of the variance in national selection first attempt score for general and vascular surgery. In addition, candidates who required more than two attempts at Part B were predicted to score substantially less at the national selection interview than those who passed at their first attempt. This difference in score would have been below previous years' minimum appointable scores, suggesting that candidates who require more than two attempts at Part B are unlikely to be offered a national training number in general or vascular surgery.

Although many candidates take several attempts to pass high‐stakes medical examinations^5^ ^17^, most studies have focused on the relationship between examination scores and future performance without distinguishing between candidates on the basis of number of attempts needed to pass, despite evidence of a relationship between number of attempts and performance indicators^15^ ^18^, ^22^. For example, McDougle and colleagues^23^ compared graduates who initially failed Step 1 of the USMLE^®^ with those who passed it first time, finding that the relative risk of not being specialty board certified was 2·2. Multiple attempts at the Medical College Admission Test (MCAT) have also been associated with an increased risk of failing Step 2 of the USMLE^®24^, whereas multiple attempts at the Professional and Linguistic Assessments Board (PLAB) test have been shown to be independently predictive of unsatisfactory training performance^25^. The relationship between the number of attempts required to pass a postgraduate UK medical examination and performance in other medical assessments is unknown, but one study^17^ found that as the number of attempts at each part of the MRCP examination increased, final passing score decreased. The present results add to the evidence that candidates who require more attempts to pass high‐stakes postgraduate medical examinations are of poorer calibre than those who require fewer attempts.

Although ethnicity was an independent predictor of the national selection score in multivariable analysis in the present study, it accounted for only 1 per cent of the variance in score, and nearly one‐quarter of ethnicity data were missing from the cohort. Reassuringly, the other non‐modifiable variables (sex, age and first language) had no effect on national selection performance and together with the ethnicity data provides further evidence that the national selection process for general and vascular surgery in the UK is both fair and unbiased.

Examination performance is not currently used as part of the selection criteria for entering specialty training in the UK. In a recent survey^26^ of US residency programme directors spanning all specialties, 93 per cent cited the USMLE^®^ Step 1 score as the most important factor in selection. USMLE^®^ scores have been shown to be related to MCAT performance, with one study^27^ finding that 17·7 per cent of the variance in Step 1 of the USMLE^®^ score was explained by MCAT performance. Given that 17 per cent of the variance in the national selection first attempt score in the present study is explained by Part B of the MRCS examination, there may be an argument for using the (currently mandatory) Part B examination as part of the selection criteria for entry into general and vascular specialty training in the UK. Given the small effect sizes and the moderate positive correlation found, it would seem sensible for other candidate factors to be considered in conjunction with Part B performance. It is also important to acknowledge that the association between MRCS performance and national selection score could be due to an inherent problem with the MRCS examination and the assessment process for national selection. The demographic of examiners, for example, is likely to be broadly similar; thus, the same unconscious bias that may occur at Part B of the MRCS examination may also apply at national selection.

This study also has implications for policy and practice. In keeping with other studies^22^, ^23^, ^24^, ^25^, these results suggest that candidates who require multiple attempts at a postgraduate medical examination are more likely to struggle with other medical assessment processes (in this case, assessment via a selection process). This information may identify doctors who will require additional support through their training and guide national bodies when considering borderline candidates or appeals. The study has implications for the number of times UK candidates should be encouraged to attempt the MRCS examination. If those requiring more than two attempts at Part B are unlikely to be offered a national training number in general or vascular surgery, this is important information for those setting out on their postgraduate medical career.

The strengths of this study are the size of the study population and that the data are from multiple cohorts and various versions of the MRCS examination. Although the examination is undertaken by more international than UK candidates, the present study focused on UK medical graduates for three reasons: UK candidates are the most homogeneous group that take the MRCS examination; the examination has been designed to assess trainees who have been through the UK training system and who are likely to continue their surgical training in the UK; and there was an available outcome measure for this group as the UK national selection process for entry into general and vascular specialty training has been shown to be reliable and fair^28^. Future research might usefully look at the whole population of those who sit the MRCS examination to compare MRCS performance across different groups. Other surgical specialty selection committees could carry out similar studies, to examine whether the MRCS examination can predict how candidates will perform in the respective national selection interviews. The study was limited by missing data for self‐declared first language (163 of 774, 21·1 per cent) and ethnicity (176 of 774, 22·7 per cent), which reduced the total number of candidates included in the multivariable analysis. Although the rate of missing data is similar to that (15–20 per cent) seen in other educational studies^29^, future research should aim to gather a more complete data set.

This study has examined only the relationship between MRCS examination performance and national selection performance for general and vascular surgery. To assess the predictive validity of the MRCS examination further it will be necessary to analyse the relationship between MRCS and other surgical training outcomes. Further research should therefore focus on the relationship between MRCS examination results and performances in clinical practice and the postgraduate exit examination that awards the diploma of Fellowship of the Royal College of Surgeons (FRCS).
